# Energy metabolic dysregulation in heat stroke: from mitochondrial dysfunction to multi-organ failure mechanisms and targeted intervention

**DOI:** 10.3389/fphar.2026.1813630

**Published:** 2026-05-13

**Authors:** Qiqi Li, Jinquan Wang, Xiaojie Qin, Biaochao Zhong, Qiantong Wei, Chunhui Zeng, Ke Yang

**Affiliations:** 1 School of Pharmacy, Guangxi University of Chinese Medicine, Nanning, China; 2 Department of Pharmacy, Maternity and Child Health Care of Guangxi Zhuang Autonomous Region, Nanning, Guangxi, China; 3 Guangxi Engineering Research Center for High-Value Utilization of Guangxi-Produced Authentic Medicinal Herbs, Guangxi University of Chinese Medicine, Nanning, Guangxi, China; 4 University Engineering Research Center of Characteristic Traditional Chinese Medicine and Ethnomedicine, Guangxi University of Chinese Medicine, Nanning, Guangxi, China

**Keywords:** energy metabolism, heat stroke, intervention strategies, mitochondria, multiple organ injury

## Abstract

Heat stroke (HS) is a life-threatening acute condition characterized by hyperthermia, central nervous system dysfunction, and multiple organ failure. Energy metabolism disruption serves as a pivotal link between hyperthermia and multi-organ injury. This review synthesizes current evidence on the mechanisms of energy metabolism dysregulation in HS and evaluates emerging intervention strategies. Mitochondrial dysfunction—manifested as structural damage, oxidative phosphorylation impairment, and excessive fission—represents an initiating event. This is followed by glucose-lipid metabolic restructuring, impaired substrate utilization, and energy depletion. These metabolic derangements mediate secondary injury in the intestine (barrier disruption and endotoxemia), brain (hypothalamic dysregulation), and lung (oxidative stress and barrier leakage). Intervention strategies are categorized into mitochondrial protection (e.g., astragaloside IV, curcumin), mitophagy modulation (e.g., melatonin, rapamycin), and substrate metabolism regulation (e.g., taurine, acetyl-L-carnitine). Notably, most current evidence for these interventions is derived from preclinical studies, and HS-validated human studies are still needed to confirm their efficacy and safety. While these approaches show promise in preclinical models, translational gaps remain, including limited validation in HS-specific models, lack of biomarker-guided patient stratification, and insufficient data on vulnerable populations. Future priorities include dynamic metabolic monitoring, identification of early-warning biomarkers, and development of personalized interventions tailored to age, comorbidity status, and metabolic phenotype.

## 1 Introduction

Heat stress refers to a series of adaptive and damaging responses triggered by increased thermal load when the body is exposed to high temperatures; it constitutes a key pathophysiological basis for the onset and progression of heat-related illnesses (Cui et al., 2025; O'Connor, 2025). Heat-related illnesses are essentially a continuous pathological process that progresses from mild adaptive imbalance to severe tissue damage. When heat stress exceeds the body’s compensatory capacity, it can develop into the most severe form of heat-related illness—heat stroke (HS) (Cui et al., 2025; O'Connor, 2025). Heat stroke (HS) is characterized by core body temperature exceeding 40 °C, central nervous system dysfunction, and multiple organ failure. It has posed a serious threat to human health, with mortality rates showing an upward trend annually (Bouchama et al., 2022; Epstein and Yanovich, 2019; Iba et al., 2025a). According to causative factors, it can be classified into classic heat stroke and exertional heat stroke (Bouchama et al., 2022). HS pathogenesis is complex, involving multiple pathways including systemic inflammatory response, coagulation dysfunction, oxidative stress, and cell death (Zhang et al., 2024). However, recent studies reveal that energy metabolism disorders occupy a central position in the development of HS, serving as the key link between hyperthermia and multi-organ dysfunction.

Under heat stress conditions, the body’s energy demand increases dramatically to cope with the physiological burden of high temperatures and repair cellular damage (Yue et al., 2024). However, prolonged exposure to high temperatures severely damages intracellular energy production systems, particularly mitochondrial structure and function, leading to inefficient energy generation. This results in a significant imbalance between energy production and consumption, plunging cells into an energy crisis that triggers cellular dysfunction and programmed cell death, ultimately manifesting as multiple organ failure (Hickey et al., 2024). Although previous reviews have extensively addressed inflammatory cascades and coagulation dysfunction in HS, a comprehensive synthesis of energy metabolism disruption—from mitochondrial dysfunction to organ-specific consequences—remains lacking. Moreover, existing intervention summaries have not critically evaluated the translational readiness of mechanism-based strategies. This review addresses these gaps by: (1) systematically analyzing the dynamic characteristics of energy metabolism disruption in HS, distinguishing adaptive from maladaptive responses; (2) delineating the mechanistic links between metabolic derangements and multi-organ injury, with focus on intestine, brain, and lung; and (3) critically evaluating emerging intervention strategies based on evidence quality, model specificity, and translational stage. By integrating these perspectives, this review aims to provide a mechanistic framework for understanding energy metabolism in HS and to identify priorities for future translational research.

## 2 Methodology

**FIGURE 1:**
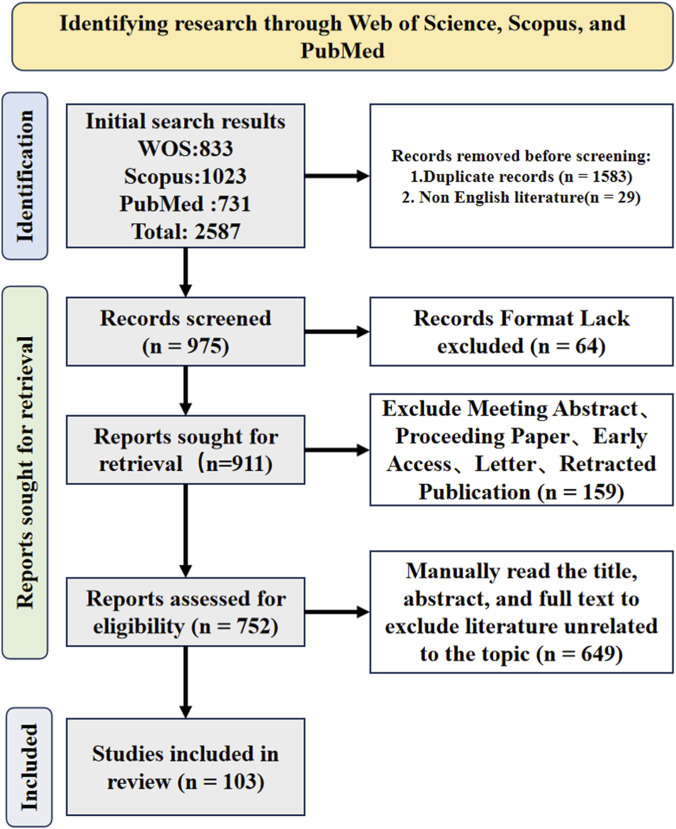
PRISMA-based flowchart of the literature search and study selection process.

In this review, we critically evaluate evidence from both heat stress and heat stroke models, explicitly distinguishing between these conditions to enhance translational relevance. This narrative review was conducted based on a literature search of PubMed, Web of Science, and Scopus databases up to January 2026 (Figure 1). The search terms included combinations of the following keywords: “heat stroke”, “heat stress”, “energy metabolism”, “mitochondrial dysfunction”, “oxidative phosphorylation”, “glucose metabolism”, “lipid metabolism”, “multiple organ injury”, “intestine”, “brain”, “lung”, and “intervention”. Articles were included if they were peer-reviewed original research or reviews written in English, with relevance to mechanistic, pathophysiological, or interventional aspects of energy metabolism in heat stroke or heat stress models. Studies focusing solely on hyperthermia without relevance to heat stroke pathophysiology were excluded (Figure 1).

## 3 Energy metabolism and supply-demand balance under heat stress

### 3.1 Overview: heat stress as a pathophysiological basis for HS energy dysregulation

Heat stroke is a clinical syndrome caused by severe heat stress. Existing research suggests that energy metabolism imbalances may be involved in its pathophysiological processes and are associated with the onset and progression of cellular damage and organ dysfunction. Given the continuous pathophysiological link between heat stress and heatstroke, exploring the mechanisms of energy metabolism disorders from the perspective of heat stress can still aid in understanding the onset and progression of heatstroke. Previous studies have shown that heat stress can induce abnormalities in key steps of cellular energy metabolism. Under conditions of heat stress, mitochondrial dysfunction may occur, manifested as decreased oxidative phosphorylation and reduced adenosine triphosphate (ATP) synthesis, accompanied by an increase in reactive oxygen species (ROS), which in turn induces oxidative stress and exacerbates cellular damage (Hickey et al., 2024). Furthermore, heat stress can disrupt the homeostasis of carbohydrate and lipid metabolism, affecting the supply and utilization of energy substrates—such as impaired fatty acid oxidation and abnormal glucose metabolism—thereby further exacerbating the imbalance between cellular energy supply and demand (Zhang et al., 2025). These changes suggest that under severe heat stress, cells may experience an imbalance between energy supply and demand, which is associated with the onset and progression of organ dysfunction; however, its specific role and causal relationship in human heatstroke require further investigation.

Energy metabolism disorders associated with heatstroke may originate from the gradual disruption of the body’s energy balance under conditions of heat stress. Therefore, clarifying the effects of heat stress on fundamental energy metabolism processes is a crucial prerequisite for understanding the metabolic abnormalities associated with heatstroke. Energy metabolism refers to the entire process by which organisms convert externally ingested nutritional substrates (glucose, fatty acids, amino acids, etc.) into directly usable high-energy molecules (ATP) through a series of coupled catabolic and anabolic reactions. This enables life activities and maintains energy homeostasis, encompassing energy release, transfer, storage, and utilization (Liu et al., 2025). During heat stress (HS), a key issue in energy supply-demand balance is whether basal energy expenditure (BEE) changes with increasing thermal load. Thus, resting energy expenditure (REE) is commonly used as a critical indicator for assessing alterations in energy requirements. It was found that heat exposure at 38 °C did not alter REE compared to 28 °C (Henkel et al., 2025). However, another controlled crossover trial on healthy people revealed a 35% increase in REE at 40 °C and 25% relative humidity relative to baseline (Henkel et al., 2025). Furthermore, a thermal environment of 50 °C and 50% relative humidity elevated REE by an additional 13% over the latter baseline (Henderson et al., 2021). It can be inferred that the impact of heat exposure on energy expenditure may correlate with the intensity of the thermal load and potentially involve a threshold effect. Alternatively, more pronounced effects on energy expenditure under heat exposure may require increasing environmental temperature, humidity load, or extending the duration of the intervention to be more clearly demonstrated.

To maintain relatively constant core body temperature, the body redistributes blood flow during heat exposure, diverting blood from core organs and skeletal muscles to cutaneous vessels to enhance heat dissipation (Millet et al., 2021). This thermoregulatory response not only alters circulatory load and tissue perfusion but may also increase the energy homeostasis required for maintaining homeostasis by affecting oxygen delivery and substrate utilization patterns, thereby providing a physiological basis for energy metabolism abnormalities. Heat exposure has been demonstrated to induce energy metabolism disorders, which are critical for maintaining cellular function. When tissue or cellular temperature rises, the rates of numerous enzymatic reactions within cells can increase exponentially, often by a factor of 2–3, thereby accelerating substrate turnover and elevating energy demand (Leon and Helwig, 2010). Under conditions of heightened energy demand and constrained supply, mitochondrial load increases, making redox balance more susceptible to disruption. Studies in heat stress models (not specific to HS) reveal that heat exposure significantly induces oxidative stress and apoptosis, suggesting limited capacity for cells to maintain normal physiological functions at elevated temperatures. Energy metabolism disruption may represent a key driver in this process (Khan et al., 2024). Therefore, the disruption of the energy balance under heat stress may represent a critical early stage in the energy metabolism disorders associated with heatstroke. Although current understanding is primarily derived from relevant experimental studies, it nevertheless provides important clues for further elucidating the pathophysiological mechanisms of heatstroke in humans.

### 3.2 Heat stress-induced mitochondrial dysfunction in HS

#### 3.2.1 Mitochondrial structural damage and inhibition of oxidative phosphorylation

As the cellular powerhouse, the functional integrity of mitochondria is crucial for sustaining cellular life activities (Ye et al., 2025). They are double-membrane-bound organelles present in most eukaryotic cells, serving as pivotal hubs for sustaining physiological functions and life processes. Their structure primarily comprises the outer membrane, inner membrane, intermembrane space, matrix, and cristae (Macherel et al., 2021). Heat production predominantly occurs on the cristae membranes, whose parallel arrangement may enhance heat storage capacity within the matrix (Lane, 2018). Energy conversion reactions predominantly occur on the inner mitochondrial membrane and cristae membranes, with a portion of energy dissipating as heat during these processes. Heat stress damages mitochondrial morphology, causing mitochondrial swelling characterized by increased volume and disruption or fragmentation of the cristae structure (Fang et al., 2024; Xiang et al., 2025).

The effects of heat stress on cells manifest as disturbances in energy metabolism, specifically through impairment of the mitochondrial oxidative phosphorylation system (OXPHOS). Mitochondria carry out energy conversion via membrane-bound enzyme complexes within OXPHOS, which comprises the respiratory chain and ATP synthase (Carlström and Ott, 2024). Under high-temperature conditions, this can lead to a reduction in mitochondrial membrane potential, inhibition of the electron transport chain (ETC), and decreased ATP synthase activity (Fang et al., 2024). OXPHOS occurs within the ETC, which comprises five polymeric protein complexes located in the inner mitochondrial membrane. These complexes transfer electrons through redox reactions, releasing energy that drives the active pumping of protons into the intermembrane space. This creates a proton gradient, providing the motive force for ATP synthase to synthesize ATP. Furthermore, heat stress disrupts electron transfer and proton pumping, compromising proton gradient maintenance and triggering energy metabolism imbalance (Skibiel et al., 2018). Studies reveal that maximum respiratory capacity and reserve respiratory capacity of C2C12 myoblasts significantly decline under high temperatures (Sajjanar et al., 2019). This indicates mitochondrial limitations in both basal energy supply and stress compensation, representing a critical component of cellular energy crisis. It is important to distinguish between adaptive and maladaptive responses: transient AMPK activation and controlled mitochondrial fission may support stress adaptation, whereas sustained activation and excessive fragmentation contribute to irreversible energy failure. This distinction has critical implications for therapeutic timing and target selection.

In addition to inhibiting ETC itself, heat stress may also reduce coupling efficiency by altering the permeability of the mitochondrial inner membrane. Proton decoupling refers to increased permeability of the mitochondrial inner membrane to protons, leading to disruption of the proton gradient (Berthole et al., 2022). Once the proton gradient is lost, ATP synthase cannot utilize the energy from proton backflow to synthesize ATP, resulting in OXPHOS decoupling (Jonckheere et al., 2012). Disruption of mitochondrial structure and function directly triggers proton leakage, subsequently causing OXPHOS uncoupling (Demine et al., 2019). Studies reveal that proton uncoupling can induce transient hyperthermia of approximately 7.5 K within seconds, indicating that the rapid collapse of mitochondrial membrane potential during heat stress itself rapidly depletes energy and exacerbates damage (Rajagopal et al., 2019). *In vitro* studies confirm that 5 min at 43 °C causes a 40% plunge in cellular mitochondrial respiratory control rate, with synchronous decline in OXPHOS and ETC, irreversible loss of membrane integrity (Mezzacappo et al., 2025), directly triggering energy depletion and metabolic disruption.

#### 3.2.2 Imbalance in the ROS-AMPK-PGC-1α axis will lead to Drp1-mediated excessive fission

Damaged mitochondria become primary sources of ROS. When ROS production exceeds cellular antioxidant defenses, oxidative stress occurs, further damaging mitochondria themselves and other macromolecules within the cell—such as proteins, lipids, and DNA—creating a vicious cycle. ROS increases nuclear expression of mitochondrial transcription factor A (TFAM) and nuclear respiratory factor 1 (NRF1), activate AMP-activated protein kinase (AMPK), and enhance the expression of peroxisome proliferator-activated receptor gamma coactivator 1-alpha (PGC-1α), a key regulator of mitochondrial biosynthesis (Irrcher et al., 2009; Kang et al., 2009). AMPK is a serine/threonine kinase comprising an α-subunit and regulatory β- and γ-subunits, forming a heterotrimeric complex (Herzig and Shaw, 2018) that serves as a key regulator of cellular energy homeostasis. When intracellular ATP levels decline, AMPK is activated and collaborates with SIRT1 (Silent Mating Type Information Regulation 2 Homolog 1) to jointly promote PGC-1α activation (Marquez Acevedo et al., 2025). The activation of PGC-1α in turn activates the Nuclear Receptor Factor (NRF) and Peroxisome Proliferator-Activated Receptors (PPARs), which regulate mitochondrial transcription factor expression to increase mitochondrial biogenesis (Liang and Ward, 2006). Moderate mitochondrial stress enhances adaptive defenses, but excessive stress proves detrimental. Although AMPK phosphorylation transiently increases during early heat stress, it subsequently becomes inhibited due to mitochondrial structural damage, further impairing cellular adaptive regulation against energy crises (Lu et al., 2017). AMPKα1/2 and β1/2 accumulate in the nucleus 2–3 h after heat stress and decrease after 5 h (Kodiha et al., 2007). A mammary epithelial cell heat stress model demonstrated that downregulated SIRT3 expression exacerbates mitochondrial fission-fusion imbalance, membrane potential decline, and reduced ATP synthesis by inhibiting AMPK phosphorylation, confirming mitochondrial dysfunction as a critical component of the energy crisis (Sun et al., 2021).

**FIGURE 2:**
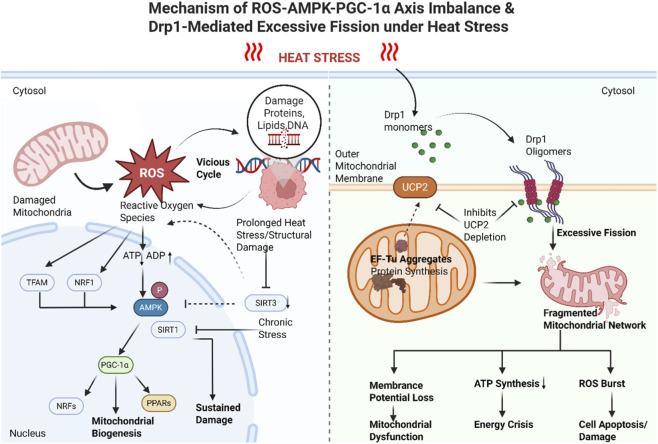
Mechanism of heat stress-induced mitochondrial dysfunction, showing mitochondrial structural damage, impaired oxidative phosphorylation, reduced ATP production, and ROS accumulation under heat stress.

Heat stress has been associated with disruption of the equilibrium between mitochondrial fission and fusion, which may contribute to mitochondrial dysfunction. While early-stage Drp1-mediated fission may represent an adaptive response to acute energy stress, sustained or excessive fission under prolonged heat stress appears to promote irreversible mitochondrial fragmentation and bioenergetic collapse, although much of this evidence is derived from cell-based or animal models and requires further validation in HS-specific context (Chen et al., 2020). Dynamin-related protein 1 (Drp1) is a cytoplasmic guanosine triphosphatase (GTPase) that exists as soluble monomers in the cytoplasm under resting conditions (Yu T. et al., 2016). Upon receiving stress signals, Drp1 is recruited to the mitochondrial outer membrane and assembles into helical oligomers, mediating membrane contraction and fission factors through GTPase hydrolysis (Yu T. et al., 2016). Under heat stress, Drp1 rapidly translocates and triggers excessive fission, leading to mitochondrial network fragmentation—an upstream driver of energy metabolism imbalance (Yu T. et al., 2016). Yu et al. demonstrated in cells that inhibiting Drp1-mediated mitochondrial fission significantly mitigates heat stress-induced loss of membrane potential and ROS bursts (Yu T. et al., 2016), suggesting that targeting mitochondrial dynamics regulation represents a potential protective strategy to alleviate the energy crisis in HS. Conversely, heat stress also induces mitochondrial protein aggregation, particularly the rapid accumulation of the translation elongation factor Tu, which impedes mitochondrial protein synthesis. This further diminishes ATP production efficiency and exacerbates mitochondrial dysfunction (Wilkening et al., 2018). Research indicates that following 43 °C heat stress, the uncoupling protein 2 (UCP2) is compensatorily upregulated to counteract thermal injury. However, depletion of UCP2 exacerbates Drp1-mediated mitochondrial fission, membrane potential depolarization, and ROS production, thereby promoting apoptosis and intensifying cellular damage (Huang et al., 2022) (Figure 2).

#### 3.2.3 Brown adipose tissue and systemic energy expenditure regulation

Brown adipose tissue (BAT) is rich in mitochondria, and its primary function is to participate in energy metabolism and thermoregulation through non-shivering thermogenesis mediated by uncoupling protein 1 (UCP1). UCP1 is a key marker molecule of brown adipocytes and plays a crucial role in the differentiation, development, and thermogenesis of BAT (Zhang et al., 2016). In rodents, BAT is rich in mitochondria, and high UCP1 expression facilitates substrate oxidation and rapid energy release. Existing studies indicate that heat stress can influence the morphological characteristics and transcriptional profiles of BAT. Under high temperature, BAT undergoes significant morphological and gene expression changes. Studies in mice reveal that under high temperatures, brown adipocyte lipid droplets expand and aggregate into unilocular droplets, shifting their morphology and gene expression profiles toward characteristics similar to white adipose tissue (WAT) (Shinde et al., 2021). Although these findings suggest conserved metabolic responses across species, direct application to human HS requires cautious interpretation given species-specific differences in metabolic rate and thermoregulation. This adaptation suggests BAT may adjust morphologically to environmental changes, exhibiting energy storage features akin to WAT.

Mitochondrial autophagy is crucial for the selective degradation of damaged mitochondria, contributing to the maintenance of mitochondrial and cellular homeostasis. Studies indicate that under thermal stress conditions, BAT selectively degrades the metabolic regulator PGC-1α via chaperone-mediated autophagy pathways, thereby suppressing mitochondrial thermogenic function (Zhuang et al., 2025). This mechanism offers a novel molecular perspective on how elevated environmental temperatures trigger energy imbalance. Further studies indicate that the thermogenic function of BAT and beige adipose tissue is also regulated by cytokines such as interleukin-6 (IL-6) and interleukin-15 (IL-15), which may influence adipose tissue thermogenesis by modulating UCP1 activity (García et al., 2018). It should be noted that the evidence regarding BAT morphological remodeling, autophagy regulation, and changes in thermogenic function cited above is primarily derived from studies in rodents, such as mice. Given the significant differences between mice and humans in terms of the anatomical distribution, tissue abundance, and thermogenic contribution of BAT—for example, BAT in mice is primarily concentrated in the interscapular region, whereas in adults it is mostly scattered (Börgeson et al., 2022)—the results of existing animal experiments cannot yet be directly extrapolated to the mechanisms underlying the onset and progression of human heatstroke. Consequently, the specific role of BAT in human heatstroke and its clinical significance remain to be confirmed by further clinical and translational studies.

### 3.3 Heat stress-induced glycolipid metabolic restructuring and imbalance in energy substrate supply

#### 3.3.1 Abnormal glucose metabolism

The oxidation of substrates generates a proton motive gradient, typically driving the phosphorylation of adenosine diphosphate (ADP) into ATP. However, heat stress reduces the efficiency of mitochondrial ATP production and induces uncoupling of oxidation and phosphorylation (Tardo-Dino et al., 2019). A single glucose molecule yields only two ATP molecules via aerobic glycolysis but generates 36 ATP molecules through OXPHOS (Bouchama et al., 2017). Under heat stress, impaired mitochondrial OXPHOS forces cells to rely on inefficient glycolysis for ATP production, resulting in overall energy insufficiency (Fang et al., 2024; Su et al., 2026). To compensate for this metabolic deficit, cells activate gluconeogenesis to synthesize glucose from non-carbohydrate sources. Studies reveal that heat stress impairs endothelial function, increasing vascular permeability and disrupting microcirculation. This further restricts oxygen and nutrient delivery to tissues, hindering aerobic energy metabolism and forcing cells toward anaerobic glycolysis, resulting in lactic acid accumulation (Iba et al., 2025b). To compensate for glycogen depletion and inadequate carbohydrate intake, the body accelerates the utilization of protein reserves. Studies in broilers indicate that prolonged heat exposure downregulates citrate synthase expression and enhances carbonyl modification of glycolytic enzymes like lactate dehydrogenase, forcing skeletal muscle to switch to inefficient anaerobic ATP production and exacerbating overall energy deficits (Liu et al., 2023). Further research indicates that extreme dry heat can suppress OXPHOS genes in peripheral blood cells even before significant core temperature elevation (Bouchama et al., 2017). This implies that mitochondrial OXPHOS impairment initiates the early energy crisis during heat stress. In healthy young males exposed to heat, elevated serum glucose and insulin levels indicate environmental temperature disrupts glucose homeostasis (Faure et al., 2016).

#### 3.3.2 Lipid metabolic disorders

When energy demands increase, lipids in adipose tissue are utilized through lipolysis to generate energy. Heat stress or exposure to high temperatures can disrupt lipid metabolism, leading to abnormal lipid mobilization, reduced fatty acid oxidation, and intracellular lipid accumulation (Wen et al., 2024). Fatty acids are activated into acyl-CoA by acyl-CoA synthetase (ACS). Subsequently, it is transferred to the inner mitochondrial membrane via carnitine acyltransferases 1 and 2, undergoing β-oxidation in the mitochondrial matrix. This process breaks down fatty acids into CO_2_ and H_2_O while releasing substantial energy. When lipid metabolism is disrupted and fatty acids cannot be metabolized efficiently, some are esterified into triglycerides, leading to intracellular lipid accumulation. Chronic triglyceride excess induces excessive lipid oxidation, generating surplus free radicals that cause oxidative damage (Szrok-Jurga et al., 2023). Heat stress, as an adaptive physiological response to exposure to high temperatures, has a significant impact on lipid metabolism. Arachidonic acid (AA) is an omega-6 polyunsaturated fatty acid found in the phospholipids of cell membranes. Heat stress can induce mitochondrial electron transport chain (ETC) dysfunction by upregulating AA levels, manifested as decreased expression of complexes I, II, and V, loss of membrane potential, and reduced oxygen consumption, thereby exacerbating oxidative stress and autophagy processes (Hu et al., 2024).

Previous studies have suggested that heat exposure can trigger a significant lipid metabolic response. A multi-omics study on acute heat exposure in humans found that after 30 min of exposure to 50 °C, plasma levels of free fatty acids, acylcarnitines, and certain sphingolipid-related metabolites increased, while levels of certain amino acids decreased, suggesting enhanced lipid mobilization and reprogramming of energy substrate utilization under acute heat exposure (Wen et al., 2024). In high-temperature exercise experiments, exercise intensity significantly modulates substrate oxidation patterns under heat stress. When ambient temperatures rise to 34 °C–40 °C, particularly during exercise of moderate to high intensity, carbohydrate oxidation increases while fat oxidation decreases relatively, suggesting that a shift toward carbohydrate utilization is more likely to occur when thermal stress is combined with exercise stress (Maunder et al., 2020). Another incremental cycling test showed that 36.3 °C reduces maximal fat oxidation intensity from 43% to 38% of maximal oxygen consumption, the total fat oxidation rate also decreased, indicating that high-temperature conditions can inhibit overall fat oxidation capacity (Ruíz-Moreno et al., 2021). Other studies in healthy individuals have shown that high-temperature environments can affect energy metabolism and oxygen utilization during high-intensity exercise (Geng et al., 2025). These findings, derived primarily from exercise trials in healthy subjects under high-temperature conditions, indicate that although lipid metabolism disorders under heat stress exhibit a certain degree of universality, they cannot be directly extrapolated to the specific metabolic processes of patients with exertional heatstroke; therefore, further clinical validation is required.

In female mice 9–14 days post-exertional HS, free fatty acids, ceramides, and diacylglycerols accumulated significantly, indicating delayed lipotoxic metabolic restructuring in peripheral tissues. This is closely associated with impaired mitochondrial β-oxidation and substrate supply-demand imbalance (Laitano et al., 2020). Another critical manifestation of metabolic disruption is ferroptosis. Ferroptosis is an iron-dependent form of cell death involving iron overload and lipid peroxide accumulation. Research has shown that lactoferrin can alleviate intestinal barrier damage in both *in vitro* models of heat-stressed intestinal epithelial cells and *in vivo* models of heatstroke in mice by modulating the MAPK signaling pathway and reducing ferroptosis (Li L. et al., 2025).

#### 3.3.3 Amino acid metabolic disorders

**FIGURE 3:**
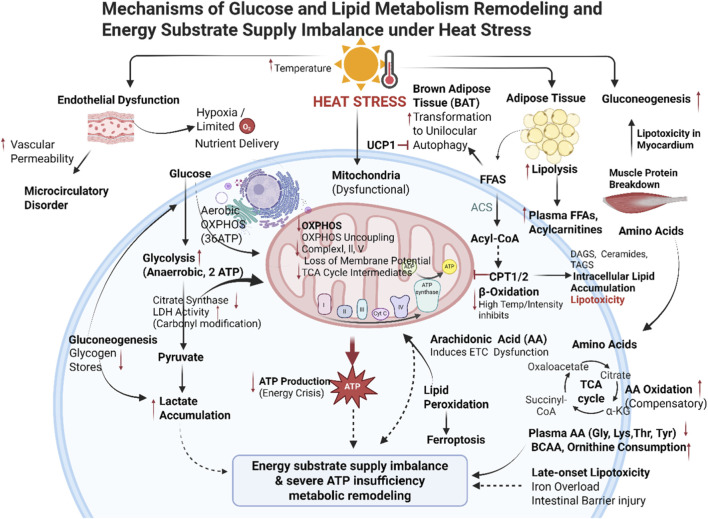
Glucolipid metabolic restructuring and imbalance in energy substrate supply under heat stress, illustrating enhanced glycolysis, reduced fatty acid oxidation, and altered amino acid mobilization.

Heat stress significantly impacts plasma amino acid concentrations. Studies reveal that heat stress markedly reduces plasma levels of glycine, lysine, threonine, and tyrosine. This change may be associated with the body activating gluconeogenesis pathways under heat stress to replenish ATP sources. Specifically, peripheral amino acids are mobilized to the liver, promoting gluconeogenesis to maintain energy balance. However, this adaptive mechanism also exacerbates energy metabolism imbalance, compelling cells to accelerate amino acid oxidation to sustain the Tricarboxylic Acid (TCA) cycle (Ma et al., 2021). Concurrently, heat stress significantly increases consumption of branched-chain amino acids (BCAAs) and ornithine, contributing to energy substrate depletion and accelerated amino acid oxidation (Kawano et al., 2024). BCAAs serve not only as essential components for protein synthesis but also play critical roles in energy metabolism. Under stress conditions, they are prioritized for oxidation as alternative energy sources. Glutamate can be deaminated by glutamate dehydrogenase to form α-ketoglutarate, which directly enters the TCA cycle to provide cellular energy. Studies have shown that after 24 h of heat exposure at 40.5 °C, TCA cycle intermediates and glutamate levels significantly decrease, indicating suppressed mitochondrial OXPHOS and insufficient ATP production (Araújo et al., 2019) (Figure 3).

## 4 Energy metabolic imbalance induced by HS

The energy metabolic disorder caused by HS is not confined to the cellular level. Instead, through complex pathophysiological mechanisms, it broadly mediates damage to multiple vital organs throughout the body, ultimately leading to multiple organ dysfunction syndrome.

### 4.1 Multi-organ damage mediated by energy metabolic dysregulation: focus on intestine, brain, and lung

#### 4.1.1 Gut: energy crisis triggers barrier dysfunction

The gut is one of the earliest organs affected in HS, and the integrity of its barrier function is crucial for maintaining internal homeostasis. Heat stress induces morphological alterations in the gut, such as significant reductions in small intestinal length and width. The intestinal barrier plays a critical role in immune protection by preventing bacteria, endotoxins, and other harmful substances from entering the bloodstream. Consequently, functional impairment of intestinal epithelial cells severely compromises health. Under conditions of heat stress and energy crisis, insufficient energy supply to intestinal epithelial cells may lead to impaired tight junctions and increased intestinal permeability, known as “leaky gut.” This disruption of barrier function allows bacteria, endotoxins, and other harmful substances from the gut to enter the bloodstream, triggering systemic inflammatory responses and endotoxemia, thereby exacerbating multi-organ damage. It is worth noting that this disruption of the intestinal barrier is more pronounced in aged hosts. Roy et al. found that periodic heat exposure can cause more pronounced increases in intestinal permeability, endotoxemia, and inflammatory cytokines, as well as T-cell immunosenescence in aged mice; in the context of infection, the aged heat-stressed group exhibited more severe disruption of the tight junction protein occludin, intestinal inflammation, and systemic inflammatory responses (Roy et al., 2025), suggesting that aging, microbiome dysbiosis, and immunosenescence collectively determine the injury threshold and prognosis following heat stress.

**FIGURE 4:**
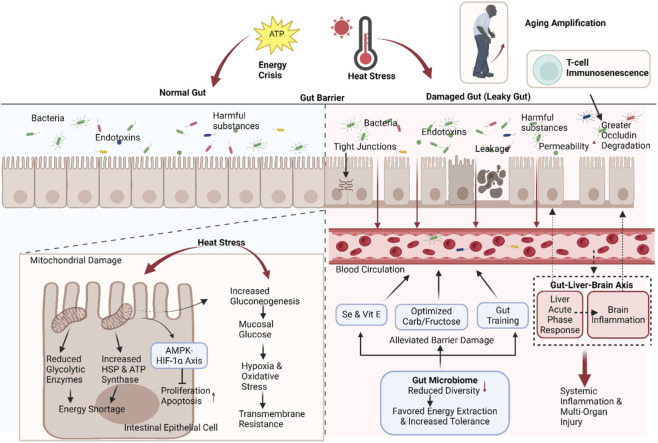
Mechanism of heat stress-induced intestinal injury, showing impaired epithelial energy supply, tight junction disruption, increased intestinal permeability, endotoxin translocation, aging amplification and gut-liver-brain axis.

It has been verified that heat stress enhances gluconeogenesis, leading to elevated glucose concentrations in the jejunal mucosa (Koch et al., 2021). This alteration may further impact energy metabolism in intestinal epithelial cells, characterized by decreased jejunal glycolytic enzymes and cytoskeletal proteins alongside increased mitochondrial ATP synthase and heat shock proteins, with energy deficiency driving barrier disruption (Koch et al., 2021). Heat stress also directly damages the barrier by inhibiting jejunal epithelial cell proliferation and accelerating apoptosis through the AMPK-HIF-1α axis (He et al., 2018). Heat stress-induced intestinal hypoxia and elevated oxidative stress also significantly reduce transmembrane resistance, increasing barrier permeability. Supplementation with selenium and vitamin E enhances glutathione peroxidase activity, thereby mitigating heat stress-induced barrier disruption (Liu et al., 2016). During heat stress, exogenous carbohydrate oxidation rates decrease by 20% (Mougin et al., 2025), suggesting the need to optimize glucose-fructose ratios and gut conditioning to improve energy supply. High temperatures reduce gut microbiota α-diversity, and these microbial changes favor energy extraction while enhancing host tolerance to extreme temperatures (Wang Z. et al., 2024) (Figure 4). Beyond local barrier disruption, heat stress-induced gut dysbiosis and increased intestinal permeability promote systemic endotoxemia and immune activation, thereby amplifying multi-organ injury. This gut-centric cascade positions the intestine as a key amplifier of systemic inflammation and metabolic dysregulation in HS (Roy et al., 2025).

#### 4.1.2 Nervous system: energy deficiency and neurotransmitter imbalance trigger central dysfunction

Thermal stress induced by high-temperature exposure often significantly impacts nervous system function, particularly in energy metabolism. Following 2 h of 43 °C exposure, rat central neurons exhibited biphasic changes in mitochondrial membrane potential: initial depolarization followed by brief recovery, then irreversible secondary depolarization. Concurrently, oxygen consumption rate peaked within 30 min before declining and remaining persistently below 50% (White et al., 2012). This alteration indicates that early-stage heat stress can induce mitochondrial respiratory chain suppression and membrane potential collapse, thereby restricting cellular energy supply. Energy deficiency not only compromises mitochondrial function but also profoundly impacts regulatory mechanisms in critical brain regions like the hypothalamus. As the primary thermoregulatory control center in the brain, the hypothalamus coordinates heat production and dissipation through diverse receptors and neurotransmitters (McCafferty et al., 2017). Rotimi et al. found that heat stress reduces hypothalamic gonadotropin-releasing hormone and pituitary luteinizing hormone and follicle-stimulating hormone synthesis, indicating that energy metabolism disruption can broadly affect central regulatory peptide synthesis, thereby exacerbating thermoregulatory imbalance (Rotimi et al., 2025). This energy deficiency not only impacts intracellular metabolic processes but may also interfere with normal central nervous system regulation, particularly thermoregulatory function.

**FIGURE 5:**
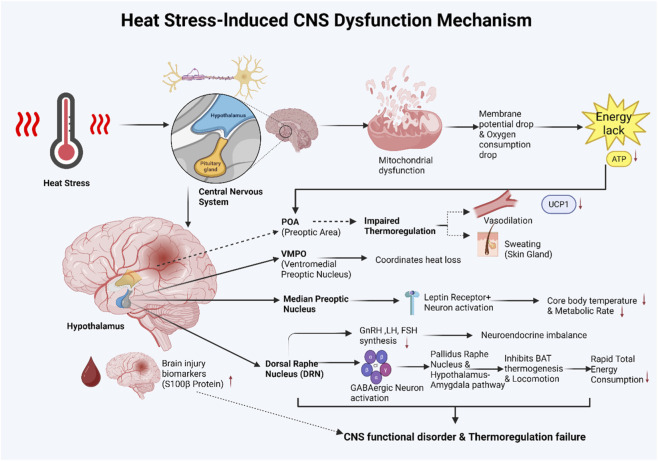
Mechanism of heat stress-induced central nervous system injury, illustrating mitochondrial dysfunction, neuronal energy crisis, and possible involvement of thermoregulatory and neurotransmitter-related pathways.

Preoptic area (POA) is the most critical region involved in autonomic thermoregulatory mechanisms (Siemens and Kamm, 2018). Neurons in this area serve as a key integrative center, receiving information from thermosensors throughout the body to maintain constant body temperature. This information is transmitted via the dorsal horn of the spinal cord or the vagus nerve, reaching the POA through polysynaptic pathways originating in the lateral parabrachial nucleus (Ambroziak et al., 2025). After POA activation, thermoregulatory responses include vasodilation, increased sweating and respiration, and inhibition of UCP1 in BAT, combined with specific behavioral responses to reduce heat production and enhance heat loss (Ambroziak et al., 2025). Within the POA, a distinct neuronal population located anterior to the ventromedial preoptic nucleus (VMPO) receives input from cutaneous thermoreceptors (Morrison and Nakamura, 2019). These thermoactivated VMPO neurons coordinate primary heat dissipation mechanisms. Leptin, a hormone produced by adipose tissue, plays a crucial role in regulating energy homeostasis. Within the median preoptic nucleus, leptin receptor-positive neurons, once activated, induce core body temperature reduction, decreased metabolic rate, and are associated with body extension behavior. This posture is commonly observed during heat adaptation in high-temperature environments (Yu S. et al., 2016). Dorsal raphe nucleus GABAergic neurons activated by heat simultaneously inhibit BAT thermogenesis and locomotion via descending pallidum-raphe and ascending hypothalamus-amygdala pathways. This process rapidly reduces total energy expenditure, representing a potential trigger for dysregulation during heat stress (Schneeberger et al., 2019). When heat stress continues to intensify and exceeds the body’s compensatory capacity, it may progress to heatstroke, accompanied by significant central nervous system damage. Existing clinical studies suggest that patients with heatstroke may exhibit elevated levels of biomarkers associated with brain injury; among these, elevated levels of S100 calcium-binding protein B (S100B) may serve as a potential predictor of poor prognosis, although evidence remains limited at this stage (Chun et al., 2019) (Figure 5).

#### 4.1.3 Lung: energy deficits trigger oxidative stress amplification and lung barrier leakage

**FIGURE 6:**
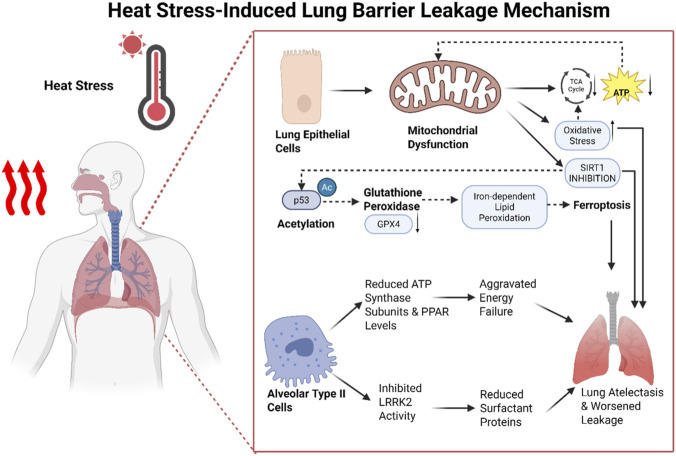
Mechanism of heat stress-induced lung injury, showing mitochondrial damage, impaired ATP production, oxidative stress, and pulmonary barrier dysfunction.

For every 1 °C increase in ambient temperature, forced vital capacity (FVC) decreases by 1.07% (Miao et al., 2022). This indicates that lung function is significantly affected by temperature fluctuations. As temperatures rise, pulmonary energy metabolism progressively becomes imbalanced, triggering a series of cellular dysfunctions—particularly alterations in mitochondrial function. SIRT1, a mitochondrial NAD + -dependent deacetylase, plays a crucial regulatory role. However, under heat stress conditions, SIRT1 function is suppressed due to mitochondrial energy metabolism disruption, further reducing TCA cycle and ATP production, thereby exacerbating cellular energy crisis. Chen et al. demonstrated that in a HS-induced lung injury model, disrupted energy metabolism in pulmonary epithelial cells causes mitochondrial membrane rupture and cristae disorganization, subsequently inducing iron-dependent lipid peroxidation. Activating SIRT1 reverses p53 acetylation, restores glutathione peroxidase expression, and mitigates pulmonary barrier leakage (Chen et al., 2022). This finding reveals SIRT1’s crucial role in regulating oxidative stress and ferroptosis, while inhibiting SIRT1-p53 activation of ferroptosis amplifies heat stress-induced damage to the pulmonary barrier. Heat exposure also reduces ATP synthase subunits and peroxisome proliferator-activated receptor levels in alveolar type II cells, inhibits leucine-rich repeat kinase II activity, and consequently exacerbates energy depletion and surfactant reduction, worsening atelectasis and leakage (Wang Y. et al., 2024). This demonstrates that energy metabolism imbalance in the lungs under heat stress not only affects cellular function but also exacerbates the severity of lung injury through multiple mechanisms (Figure 6).

## 5 Targeted intervention strategies for energy metabolism

In recent years, research on HS has increasingly focused on alleviating symptoms and improving pathophysiological conditions through the regulation of energy metabolism. Related targeted intervention strategies have gradually become a hot topic in research. Intervention strategies are categorized below based on mechanism of action, model specificity (HS versus general heat stress), intervention timing (prophylactic versus therapeutic), and translational stage. A summary table is provided to facilitate critical appraisal (Table 1).

**TABLE 1:** Summary of targeted interventions for energy metabolism disorders.

Intervention category	Intervention/Agent	Mechanisms of action	Model type	Model species	Timing of intervention	Translational research phase
Mitochondrial targeting, protection, and homeostasis restoration	Astragaloside IV	Inhibits excessive MCU activation and maintains calcium homeostasis	Heat-stress-specific model (heat inhalation injury/heat-induced bronchial epithelial cell apoptosis model)	*In vitro*: Human bronchial epithelial cell line 16HBE14o-; In vivo: Wistar rats	*In vitro*: Administered 6 h after heat treatment at 47 °C; In vivo: Administered via gavage twice daily following heat inhalation injury in rats	Basic research (In vitro cell studies + *in vivo* animal experiments)
​	Curcumin	Regulates redox balance and promotes mitochondrial fusion/biogenesis	Heat-stress-specific model	*In vitro*: Mouse C2C12 myoblasts; Ex vivo: Rat flexor digitorum brevis (FDB) muscle fibers; In vivo: C57BL/6J mice	*In vitro*: Pre-treated 30 min prior to heat exposure (43 °C); In vivo: Administered via gavage daily at doses of 15–100 mg/kg for 10 consecutive days prior to heat exposure	Basic research (In vitro cell studies + *ex vivo* muscle fiber studies + *in vivo* animal experiments)
​	Resveratrol	Activates the Nrf2 pathway and enhances antioxidant defense	Heat-stress-specific model (high-temperature group: 33 °C ± 1 °C, 10 h/day) compared with a standard heat-stress model (normal-temperature group: 22 °C ± 1 °C, 24 h/day)	*In vivo*: 21-day-old male Cobb broiler chickens	Add 400 mg/kg to the diet starting at 21 days of age for 21 days, concurrently with heat treatment	Basic research (In vivo animal experiments)
​	Green tea polyphenols	Activates the Nrf2 pathway and upregulates antioxidant genes	Heat stress-specific model	Japanese quail (*Coturnix coturnix* japonica, female, 5 weeks old)	Add to the diet throughout the entire period (200, 400 mg/kg) for 12 weeks, concurrently with heat treatment	Basic research (In vivo animal experiments)
​	Curcumin nanoparticles	Strengthens the Nrf2-SOD pathway for antioxidant defense	Heat stress-specific model	Ross-308 broiler chickens	Begin at 1 day of age; continue intervention during heat stress exposure until 35 days of age	Basic research (In vivo animal experiments)
​	Astaxanthin	Stabilizes mitochondrial membrane potential and inhibits fragmentation	Heat stress-specific injury model	Mice (C2C12 myoblasts), rats (flexor digitorum brevis muscle fibers)	Pre-treatment 30 min before heat stress (43 °C); continuous intervention throughout	Basic research (In vitro cell studies + *ex vivo* muscle fiber studies)
Regulation of mitochondrial autophagy	Melatonin	Scavenges mitochondrial ROS, stabilizes mitochondrial membrane potential, reduces lipid peroxidation and DNA damage, and inhibits apoptosis	Heat stress-specific model	Human sperm	Pre-treatment 30 min before heat stress	Basic research (In vitro studies)
​	Rapamycin	Inhibits mTOR, activates the Pink1/Parkin pathway, promotes mitochondrial autophagy, reduces ROS and MDA, and alleviates hypothalamic damage	Heatstroke model	Rats (male SD rats)	Intraperitoneal injection for 7 consecutive days prior to modeling	Basic research (In vivo animal experiments)
​	Shengmai San	Activate the AMPK/Drp1 pathway to promote mitochondrial autophagy; regulate glycolysis and the TCA cycle to improve energy metabolism; provide antioxidant and anti-inflammatory effects; and protect against liver damage	Heat stress-specific model	Rats (SD rats)	Gastric gavage 2 h before daily heat exposure for 7 consecutive days	Basic research (In vivo animal studies)
Substrate metabolism and regulation of energy compounds	Taurine	Promote lipolysis; regulate protein metabolism; inhibit the expression of genes associated with muscle atrophy; enhance protein synthesis; and improve lipid metabolism	Heat stress-specific model	Broiler chickens (male Arbor Acres)	Continuous intervention during heat stress	Basic research (In vivo animal studies)
​	Bitter melon extract	Reduces serotonin and prolactin, increases dopamine, accelerates ammonia metabolism, and alleviates central and peripheral fatigue	Heat stress-specific model	Human	Continuous intervention during high-temperature training; supplementation before, during, and after training	Preliminary translational research in humans
​	Acetyl-L-carnitine	Alleviates oxidative stress, upregulates autophagy, stabilizes mitochondrial membrane potential, and inhibits apoptosis	Heat stress-specific model	Mouse spermatogonia (GC-1)	Administered immediately after heat stress (42 °C, 90 min)	Basic research (In vitro cell studies)
​	Mung bean polyphenols	Downregulates HSP70, modulates the FoxO/MAPK/PI3K-Akt pathways, improves lipid and energy metabolism, and inhibits apoptosis	Heat stress-specific model (39/41/43 °C In vitro heat injury model)	Mouse small intestinal epithelial cells (Mode-k cells)	Pre-treated 12 h before heat stress	Basic research (In vitro cell studies)
Other	Ephedra + gypsum extract	Inhibits hypothalamic HSP70, NF-κB, and IL-1β; lowers body temperature; reduces weight loss; alleviates hypothalamic inflammation	Heat stress-specific model	Mouse (ICR mice)	Administered via daily gavage for 3 consecutive days, with intervention prior to heat exposure	Basic research (In vivo animal studies)
​	Niacin	Antioxidant effects; promotes heat dissipation and cooling; inhibits apoptosis; restores Cx43 and hormonal homeostasis	Heat stress-specific model	Mouse (BALB/c mice)	Continuous intervention during heat stress	Basic research (In vivo animal studies)
​	Coenzyme Q10	Enhances antioxidant defense, inhibits apoptosis and inflammation, and promotes androgen synthesis	Heat stress-specific model	Rat (Wistar rats)	Administration began prior to heat stress, once every other day, for 8 weeks	Basic research (In vivo animal studies)
​	Surface wetting	Reduces core body temperature and cortisol levels; inhibits hypothalamic HSP, HSF4, and AMPK activation	Heat stress-specific model	Broiler chickens (Cobb 500)	Concurrent intervention during heat stress	Basic research (In vivo animal studies)
​	Heat acclimatization	Upregulates and enhances the function of TRPV1 thermosensitive neurons in the medial preoptic area (mPOA); inhibits thermogenesis and promotes vasodilation via the mPOA→DMH/RPa circuit, thereby improving heat stress tolerance	Heatstroke model	Mouse (male C57BL/6; TRPV1-Cre)	Heat acclimatization training for 14 consecutive days prior to modeling	Basic research (In vivo animal studies)

### 5.1 Mitochondrial targeted protection and homeostatic restoration

During HS, mitochondrial calcium homeostasis disruption and oxidative stress surges constitute the pivotal mechanisms triggering energy metabolism collapse. Consequently, targeted regulation of mitochondrial calcium homeostasis and redox balance emerges as a critical intervention strategy for restoring cellular energy supply and mitigating heat injury. The mitochondrial Ca^2+^ uniporter (MCU) serves as the primary channel regulating mitochondrial calcium homeostasis. Its excessive activation leads to calcium overload, ATP depletion, and oxidative stress—changes that play a pivotal role in the energy crisis of HS. To block this pathological process, Dong et al. discovered that astragaloside IV effectively maintains energy homeostasis in bronchial epithelial cells and inhibits apoptosis by suppressing excessive MCU activation. This action mitigates heat stress-induced Ca^2+^ overload, ATP depletion, and reactive oxygen species (ROS) bursts (Dong et al., 2017). These findings provide crucial evidence for mitochondrial-targeted therapies in treating HS-induced lung injury.

Beyond regulating calcium homeostasis, heat stress directly impairs mitochondrial fusion and biogenesis, leading to mitochondrial dysfunction. Yu et al. discovered that curcumin activates NADPH oxidase to induce moderate ROS, which serves as an adaptive signal triggering mitochondrial quality control programs. This promotes mitochondrial fusion and biogenesis, thereby alleviating heat stress-induced mitochondrial dysfunction and cellular damage (Yu et al., 2020). This reveals a novel strategy for initiating mitochondrial self-repair through mild oxidative stress.

Oxidative stress is another critical factor impairing mitochondrial function, and restoring the antioxidant defense system is one of the core objectives for mitochondrial protection. Resveratrol alleviates oxidative stress and restores antioxidant enzyme expression by activating the Nrf2 signaling axis. This contributes to restoring redox homeostasis in immune organs, thereby reducing mitochondrial ROS load and favoring the maintenance of respiratory chain function and membrane potential stability (Zhang et al., 2018). This provides a novel intervention strategy for enhancing mitochondrial antioxidant capacity in immune organs affected by HS. Additionally, Khan et al. found that green tea polyphenols can upregulate antioxidant gene expression—including superoxide dismutase, catalase, and glutathione peroxidase—via the Nrf2 pathway, thereby mitigating mitochondrial oxidative damage (Khan et al., 2025; Sahin et al., 2010). This protects respiratory chain complex activity and membrane potential, supporting ATP production efficiency, confirming the feasibility of protecting mitochondrial energy metabolism by activating endogenous antioxidant pathways. However, regarding the intrinsic antioxidant defense mechanisms of mitochondria under acute heat stress, Miao et al. found that although acute heat stress increases intracellular ROS levels, it simultaneously activates the Nrf2 pathway, enabling short-term maintenance of mitochondrial redox homeostasis (Miao et al., 2020). Curcumin nanoparticles further enhance this antioxidant effect via the Nrf2-SOD pathway, significantly mitigating severe heat stress-induced hepatic mitochondrial damage (Abdel-Moneim et al., 2025). This not only highlights the pivotal role of activating endogenous antioxidant defenses in mitochondrial function protection but also provides crucial experimental evidence for developing mitochondria-targeted nanomedicines. Notably, while curcumin shows protective effects in some preclinical models, a clinical study in humans undergoing exertional heat stress reported that dietary curcumin supplementation did not alter AMPK or SIRT1 expression in peripheral blood mononuclear cells (Falgiano et al., 2018), suggesting that translation to human HS may require optimized formulations, dosing, or combination strategies. This discrepancy highlights the importance of validating preclinical findings in human-relevant models and underscores the translational gap currently limiting clinical application.

Additionally, maintaining stable mitochondrial dynamics is crucial for protecting energy metabolism. Astaxanthin safeguards ATP production in skeletal muscle under heat stress by stabilizing mitochondrial membrane potential, inhibiting excessive mitochondrial fragmentation, and upregulating the PGC-1α/TFAM signaling pathway (Yu et al., 2019), thereby offering a novel mitochondrial-targeted strategy to alleviate skeletal muscle energy depletion in HS. Therefore, by inhibiting abnormal Ca^2+^ influx, regulating redox balance, and stabilizing membrane potential alongside mitochondrial dynamics/biogenesis, mitochondrial function can be protected under heat stress conditions, restoring energy metabolism and reestablishing cellular energy homeostasis. This series of studies lays a solid experimental foundation for developing targeted interventions for HS.

### 5.2 Mitochondrial autophagy and damage repair

During HS, the accumulation of damaged mitochondria exacerbates oxidative stress and energy metabolism disorders. Therefore, timely clearance of abnormal mitochondria and restoration of mitochondrial network homeostasis are critical intervention strategies to reverse the energy crisis. Melatonin, as a natural mitochondrial protector, primarily targets heat stress damage by regulating mitochondrial autophagy. It stabilizes Parkin-mediated mitochondrial autophagy, reducing abnormal mitochondrial accumulation to restore membrane potential, improve cellular energy status, and inhibit apoptosis (Yoon et al., 2019). Simultaneously, it mitigates heat stress-induced cellular damage by suppressing ROS production, enhancing mitochondrial membrane potential, and reducing lipid peroxidation products (Zhao et al., 2021). This offers a highly promising natural intervention strategy for mitochondrial-targeted protection against multi-organ injury in HS.

To explore broader autophagy regulatory strategies, researchers also focused on rapamycin. As an autophagy inducer, it alleviates mitochondrial membrane potential decline and oxidative stress in HS mice by inhibiting hypothalamic mTOR phosphorylation and activating Pink1/Parkin-dependent mitochondrial autophagy (Chi et al., 2025). This study reveals the role of central regulatory pathways in systemic mitochondrial quality control, providing new theoretical support for central interventions in HS.

Addressing the prominent issue of energy metabolism dysfunction in HS, research on Shengmai San focuses on restoring mitochondrial function. It alleviates energy impairment by activating AMPK-Drp1-mediated mitochondrial autophagy, thereby restoring the TCA cycle and ATP production (Zhang et al., 2022). This confirms the feasibility of traditional compound Chinese medicine in protecting organ function by regulating mitochondrial autophagy, providing experimental support for integrated Chinese and Western medicine approaches to treating HS. Consequently, modulating mitochondrial autophagy through diverse pathways effectively mitigates energy metabolism disorders induced by heat stress, offering multidimensional experimental evidence for developing targeted intervention strategies centered on mitochondrial quality control.

### 5.3 Substrate metabolism and energy substrate regulation

HS disrupts the mobilization of energy substrates and metabolic pathways, directly triggering an energy supply crisis. Therefore, targeting substrate metabolism regulation to restore energy substrate homeostasis represents a critical intervention strategy for alleviating energy depletion in HS. To reverse heat stress-induced energy substrate mobilization impairment, researchers discovered that taurine, as a key metabolic regulator, activates adipose tissue lipolysis, elevates serum free fatty acids, and enhances muscle carnitine palmitoyltransferase 1 expression, partially reversing heat stress-induced energy substrate mobilization impairment (Lu et al., 2019). This provides a novel metabolic target for correcting energy metabolism imbalance in HS.

Following taurine research, studies on bitter melon extract have focused on regulating synergistic disorders in central nervous system and mitochondrial metabolism, particularly in protecting mitochondrial function and maintaining energy metabolic homeostasis. It maintains mitochondrial function and energy homeostasis under high temperatures by activating the AMPK pathway, reducing ammonia accumulation, and balancing central serotonin and dopamine levels (Kwak et al., 2020). This reveals the synergistic role of central metabolic regulation and mitochondrial function protection, offering new insights for multi-target interventions in HS.

To enhance mitochondrial energy metabolism efficiency, Qiao et al. focused on acetyl-L-carnitine (ALC). As a naturally occurring substance in the body and an effective substrate for mitochondrial energy metabolism, ALC significantly alleviates oxidative damage and apoptosis in cells following heat stress by promoting fatty acid β-oxidation, stabilizing membrane potential, and inducing autophagy flux (Qiao et al., 2021). Its protective potential for energy-crisis organs provides experimental evidence for developing nutritional intervention strategies to enhance mitochondrial metabolic efficiency. Addressing the energy crisis in intestinal epithelial cells during HS, mung bean polyphenols focus on regulating membrane lipid metabolism to reverse energy depletion. By synergistically regulating choline and glycerophospholipid metabolism, it significantly reverses energy crisis in intestinal epithelial cells of heat-stressed mice, increasing mitochondrial membrane phospholipids and inhibiting the FoxO apoptotic pathway (Feng et al., 2024). This work offers a novel strategy for natural product intervention in energy metabolism disorders during HS and provides metabolic-level theoretical support for protecting intestinal barrier function.

### 5.4 Other approaches

Beyond the direct interventions targeting mitochondrial function and substrate metabolism described above, certain natural products and traditional Chinese medicine formulas have demonstrated potential to regulate inflammatory responses and improve redox homeostasis. These mechanisms indirectly restore energy metabolic balance, offering complementary strategies for multidimensional intervention in HS.

Addressing the synergistic effects of inflammatory storms and energy depletion in HS, research on Ephedra and gypsum focuses on alleviating inflammatory responses to protect energy metabolism. This traditional Chinese medicine combination restores energy metabolic balance to some extent by downregulating the expression of heat shock protein 70 and NF-κB (Kim et al., 2019). This confirms the value of anti-inflammatory strategies in improving energy metabolic disorders in HS, providing a new entry point for integrated Chinese and Western medicine treatment. Energy depletion in HS extends beyond systemic metabolic disorders to involve damage in energy-sensitive organs such as the testes. Zahra et al. found that nicotinic acid alleviates testicular energy depletion in heat-stressed mice by enhancing NAD(P) and SOD activity (Zahra et al., 2025), identifying a novel metabolic intervention target for protecting reproductive system function in HS. Coenzyme Q10 has been demonstrated to mitigate oxidative damage to testicular mitochondria under heat stress and restore energy metabolism (Delkhosh et al., 2021).

Beyond natural products and traditional Chinese medicine interventions, early interventions such as physical cooling and thermoregulatory adaptation offer novel pathways for protecting energy metabolism. Among early physical interventions, surface wet cooling can block hyperthermia-induced excessive activation of hypothalamic AMPK, delaying depletion of the body’s energy reserves (Rajaei-Sharifabadi et al., 2017). Thermoregulatory adaptation activates the preoptic TRPV1-mPOA-DMH/Rpa pathway, suppressing BAT thermogenesis while dilating cutaneous vasculature to rapidly enhance heat dissipation and reduce energy expenditure (Li J. et al., 2025). These findings demonstrate that diverse thermal stress interventions exhibit versatility and complementarity in energy recovery, providing a rich theoretical and experimental foundation for future targeted interventions for multiple pathways and mechanisms.

## 6 Discussion

Mitochondrial dysfunction serves as the initiating core of energy metabolism disorders in HS. Heat stress directly causes mitochondrial structural damage and functional impairment, subsequently triggering imbalances in the ROS-AMPK-PGC-1α axis, suppression of BAT thermogenesis, and restructuring of glucose and lipid metabolism, thereby forming a vicious cycle of structural, functional, and metabolic abnormalities. Energy metabolism disorders mediate severe secondary damage to multiple organs due to insufficient energy supply, ultimately leading to multiple organ dysfunction syndrome. However, certain limitations exist. (1) At the mechanistic level, the spatiotemporal dynamics of energy metabolism disorders in HS remain unclear. The time-dependent patterns of core regulatory pathways, such as AMPK activity and glycolytic pathways, require further investigation. For instance, AMPK activity exhibits bidirectional changes: it may be activated during early heat stress to initiate stress adaptation due to energy depletion, but its activity may decline later due to worsening mitochondrial damage. Additionally, cell-specific metabolic mechanisms remain unclear, with differences in metabolic responses and contributions to organ damage among various cell subtypes under heat stress yet to be elucidated. Questions regarding the activation mechanism of thermal defense responses—whether driven by the initiation of thermoreceptive pathways or the inhibition of cold afferent pathways—also require urgent resolution. (2) Technological limitations: The lack of *in vivo* mitochondrial function imaging techniques hinders real-time observation of the relationship between mitochondrial function changes and energy metabolism disorders in living organisms, thereby restricting deeper understanding of the mechanisms underlying energy metabolism disorders. (3) Clinical Translation: Existing intervention studies predominantly focus on healthy young populations. However, high-risk groups for HS (e.g., the elderly, individuals with chronic diseases) exhibit distinct metabolic profiles compared to healthy individuals, and personalized intervention protocols remain scarce. The dosage, formulation, and intervention duration of certain natural products require optimization to enhance their regulatory effects on energy metabolism-related proteins. For instance, while curcumin reduces intestinal barrier damage and systemic inflammatory markers during heat stress, it fails to alter the expression of energy metabolism-related proteins such as phosphorylated AMPK and SIRT1 in peripheral blood mononuclear cells (Falgiano et al., 2018). Additionally, the absence of effective metabolic early warning biomarkers hinders the implementation of early HS detection and risk stratification.

Host vulnerability plays a critical yet underexplored role in determining HS susceptibility and outcomes. Aging, baseline mitochondrial dysfunction, chronic metabolic diseases, immune senescence, and gut microbiome fragility collectively modulate the threshold for thermal tolerance and the severity of organ injury. Recent transcriptomic and microbiome analyses have highlighted the gut-liver-brain axis as a key mediator of heat-induced pathology in aging populations (Roy et al., 2024), and have shown that aging synergistically interacts with heat stress to enhance susceptibility to infections (Roy et al., 2025). Integrating these vulnerability factors into risk stratification and personalized intervention frameworks represents a priority for future translational research. Early detection and risk stratification remain major translational gaps in HS management. Candidate biomarkers include metabolic indicators such as plasma free fatty acids, acylcarnitines, and TCA cycle intermediates, as well as barrier-related markers like intestinal fatty acid-binding protein (I-FABP) and lipopolysaccharide-binding protein (LBP). Future prospective studies are needed to validate these candidates in HS cohorts and to integrate multi-omics approaches for early warning and prognostic stratification.

Future research should further deepen mechanistic investigations, focusing on elucidating the regulatory mechanisms of Drp1-mediated mitochondrial autophagy, brain energy metabolism plasticity, and the selective utilization mechanisms of BAT toward different energy substrates. It is also crucial to determine whether shifts in substrate utilization preferences under hyperthermic stress correlate with the progression of HS and organ damage. Future efforts should promote the application of dynamic metabolic monitoring technologies, integrating spatiotemporal metabolomics with *in vivo* mitochondrial imaging to comprehensively analyze activity changes in core energy metabolism regulators and real-time metabolic remodeling. Metabolic biomarkers for early warning should be identified in HS-prone populations, enabling early detection and risk stratification through monitoring specific metabolite levels or metabolic enzyme activities. Based on population-specific metabolic profiles, personalized intervention protocols should be developed and validated through multicenter clinical trials. Against the backdrop of global warming, elucidating the regulatory pathways of metabolic thermogenesis in response to environmental heat overload will provide systematic solutions for HS prevention and treatment.
